# Occurrence and source apportionment of Per- and poly-fluorinated compounds (PFCs) in North Canal Basin, Beijing

**DOI:** 10.1038/srep36683

**Published:** 2016-11-15

**Authors:** Yi-Zhe Zhang, Bin Wang, Wei Wang, Wen-Chao Li, Jun Huang, Shu-Bo Deng, Yu-Jue Wang, Gang Yu

**Affiliations:** 1 Beijing Key Laboratory of Emerging Organic Contaminants Control, State Key Joint Laboratory of Environmental Simulation and Pollution Control, Collaborative Innovation Center for Regional Environmental Quality, School of Environment, Tsinghua University, Beijing, 100084, China; 2 CSD IDEA (Beijing) Environmental Test&Analysis Co., Ltd., Beijing 100192, China

## Abstract

Various per- and poly-fluorinated compounds (PFCs) were first systematically investigated in North Canal Basin, Beijing, China. A total of 68 surface water samples were collected from North Canal Basin, Beijing, at high spatial resolution. The seasonal disparity was compared and associated with source variation. PFCs concentrations in low-water period ranged from 26 to 207 ng/L, and significantly declined levels were found in high-water period. The individual component proportions among different sites varied less in high-water period, when runoff played a role in mixing and diluting PFCs. A methodology combined with principal component analysis (PCA), heat map-hierarchical cluster analysis (HM-HCA), and correlation analysis were introduced to discriminate sources of PFCs in surface water. The statistical results agreed with each other, and daily domestic consumption, fire-fighting products and related industries were identified as sources of PFCs in this region. In addition, two composition ratios were proposed through the methodology to distinguish the impact of nonpoint source, and the outcome demonstrates that great disparities exist in compositional profiles between nonpoint source and others. Overall, the results showed that this comprehensive analysis method has great potential for source apportionment in surface water and other environmental compartments.

Per- and poly-fluorinated compounds (PFCs), including perfluorinated carboxylates (PFCAs), sulfonates (PFSAs) and various precursors, have been applied to industrial and household products for decades^1^,^2^. PFCs have been widely used in industry as surfactants, lubricants, fire retardants, paper and textile treatment^3^,^4^,^5^. They are water-soluble, bioaccumulative and persistent in the environment^6^. PFCs have been ubiquitously detected in abiotic and biotic media, including surface and ground water, sediments, aquatic organisms, human blood and breast milk^7^,^8^,^9^,^10^. In addition, some PFCs have been found to have acute and subchronic toxicity as peroxisome proliferators, gap junction and intercellular communication inhibitor^3^,^11^,^12^,^13^,^14^. Thus, long time exposure to PFCs would pose potential risk to human being and ecosystem^15^,^16^.

In recent years, much attention has been paid to PFCs in aqueous environments. PFCs pollution in surface water has been reported globally^17^,^18^,^19^,^20^,^21^. Generally, perfluorooctanoic acid (PFOA) and Perfluorooctane sulfonate (PFOS) were the most detected and concentrated perfluorinated compounds in surface water. The concentration of each chemical could reach the level of several hundred ng/L in river basin^5^,^22^,^23^,^24^,^25^. The contamination level often exceeds the recommended standards of PFCs for drinking water proposed by state of New Jersey (0.04 μg/L for PFOA^26^) and the German Drinking Water Commission (0.1 μg/L for PFOA plus PFOS^27^). Past studies showed that occurrence of PFCs was strongly correlated with population density and highly influenced by urban activities^28^,^29^,^30^. It is also interesting to note that higher PFCs loads in surface water have been observed during wet-weather^31^,^32^.

Generally, PFCs input into the aqueous environment occurs via four routes: (1) discharge from wastewater treatment plants (WWTP); (2) influx of runoff contaminated by nonpoint source; (3) atmospheric deposition of oxidized precursors; (4) seepage and illegal discharge. Significant variances were observed between the profiles of different sources^33^,^34^,^35^. Although quantification of PFCs in water environment has been successfully explored and applied, source identification can still be a challenging task, because PFCs were widely used and they were dispersed and underwent complex transformation. In some cases, it was handled by analyzing specific composition of all possible sources^29^,^33^. In other complicated environment system, the sources of PFCs were able to be traced by studying their spatial and temporal distribution and the ratios of discrete components. Xu *et al.*^9^ employed principal component analysis (PCA) to investigate the possible sources for sediments. Nguyen *et al.*^27^ used loading ratio method to evaluate the source of non-/point sources. Cluster analysis can help to differentiate contributions of each sources^30^. However, exploited separately, these models are not enough to provide accurate and comprehensive information of source apportionment.

Beijing is a very typical monsoon region. The maximum and minimum rainfall occurred in July and December respectively, and the former is about 100 times as much as the later. The contamination status and seasonal variation of various PFCs in Beijing, one of the most densely populated regions in the world, has not been properly studied. This study is aimed to fill in the data gap of PFCs contamination status of North Canal basin in both high-water period (HWP) and low-water period (LWP), to statistically analyze the spatial distribution pattern and seasonal variation, and to develop a methodology in order to identify sources. The method is based on multivariate data analysis, including PCA, heat map-hierarchical cluster analysis (HM-HCA), and correlation analysis, which make it easier to analyze raw data of environmental monitoring, and to track down the sources of PFCs. Besides, widely application of chlorinated polyfluorinated ether sulfonate (locally called F-53B, C_8_ClF_16_O_4_SK), a Chinese PFOS alternative, has been encouraged by Chinese government, as an important part of implementing Stockholm Convention. To the best of our knowledge, this is the first systematic investigation of F-53B together with various PFCs in a regional environment, after Wang *et al.*^36^ first reported its usage in China.

## Results and Discussion

### Concentration and composition profile of PFCs in the surface water

**Figure 1:**
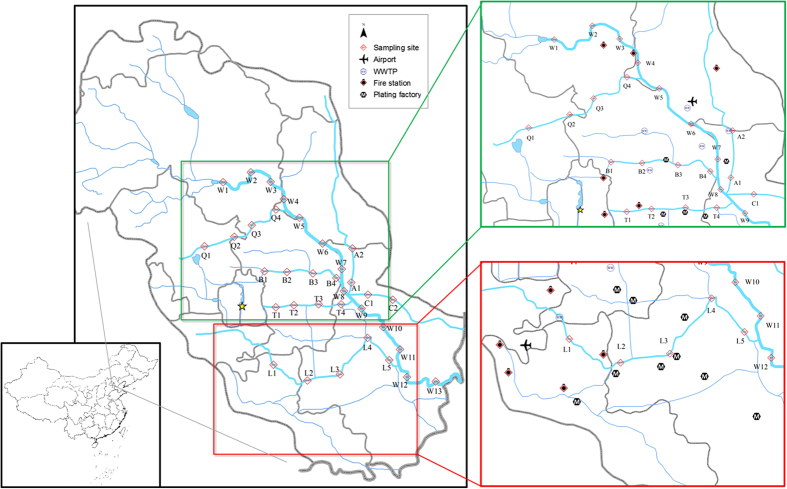
Sampling sites in North Canal and its tributaries, northeastern Beijing. (We created this figure using ArcMap 10.2, http://www.esrichina.com.cn/).

**Figure 2:**
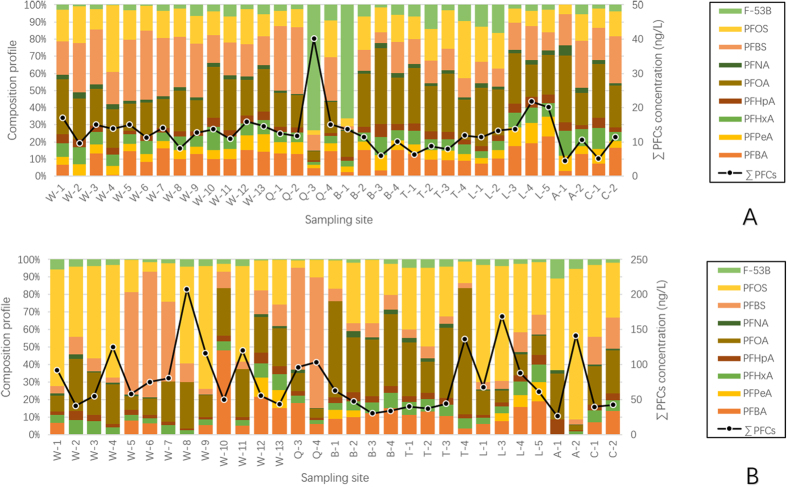
The concentrations and compositional profiles of 9 PFCs in surface water of North Canal Basin in different water periods. (**A**) HWP; (**B**) LWP.

The concentrations of ∑PFCs (the sum of 9 measured PFCs concentrations) in North Canal and its tributaries ranged from 4.49 to 40.12 ng·L^−1^ in the HWP, and 26.11 to 207.56 ng·L^−1^ in LWP. The highest ∑PFCs concentration of LWP was observed in W8, where several tributaries join the main stream. The lowest total concentration was found in A1 for both HWP and LWP. According to Figs 1 and 2 and Table S1, in both two samplings, the contamination status of Xiaozhong River (A1, A2), Yunchaojian River, (C1, C2) are lower than the North Canal, which can be explained by running through the less developed areas. Nevertheless, flowing past the highly populated and industrialized areas may account for the high ∑PFCs loadings of Qing River (Q1 to Q4) and Liangshui River (L1 to L5)^37^.

The average concentrations of individual PFCs were exhibited in Tables S4 and 5, PFOA showed the highest average concentration (2.89 ng·L^−1^) in the HWP, followed by PFBS (2.61 ng·L^−1^), PFOS (1.94 ng·L^−1^), while PFOS, PFOA and PFBS predominated in the LWP, with the average concentrations of 32.17 ng·L^−1^, 16.82 ng·L^−1^, and 12.57 ng·L^−1^, respectively. Together the three compounds took up 16.98–80.41% (median of 64.34%) and 40.95–92.56% (78.85%) of total PFCs concentration in HWP and LWP, respectively. Most of the compounds had been identified and peaked in Liangshui River (the biggest municipal drainage channel of Beijing, Fig. 1). The consequence is verified by a previous report that seven discharge outlets along the Liangshui River discharged 60,000 tons of untreated wastewater into the river per day^38^.

**Figure 3:**
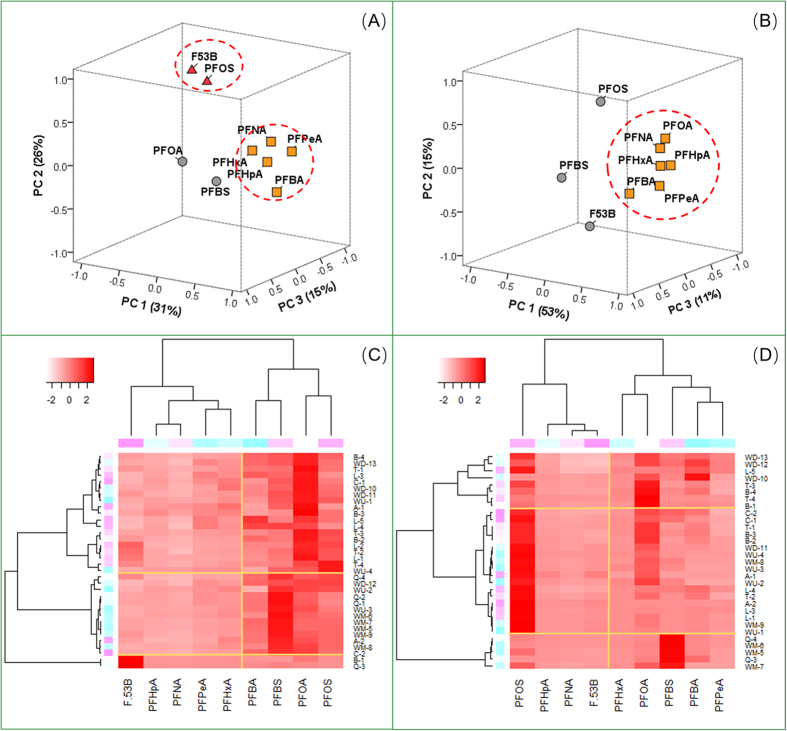
PCA and HM-HCA results of PFCs in two water periods. (**A**) HWP Component Plot in Rotated Space; (**B**) LWP Component Plot in Rotated Space; (**C**) two-dimention HM-HCA of HWP, for 31 sites (right of the map) and 9 PFCs (bottom of the map); (**D**) HM-HCA for LWP, for 34 sites and 9 PFCs. In Heat map, the color gradient represents the concentration intensity from the lowest (−3) to the highest (3). (More interpretation of the references to HM-HCA and PCA models were listed in the supplementary information).

In LWP, the compounds were spotty distributed (Figs 2 and 3), suggesting the influence of point discharges^30^,^39^, while in HWP, the distribution was less fluctuated (Supplementary Tables S4 and 5, Fig. S2), whereas an overall concentration decline was clearly observed. Such circumstance might result from the mixing and diluting effects of large amount runoff during continuous wet weather^27^,^31^,^32^,^35^. Particularly, in HWP, the percentage of PFOS was significantly decreased, while the share of several other PFCs (PFBA, PFPeA, PFHxA, PFHpA, PFOA and F-53B, Fig. 2) were clearly elevated. The comparison suggested that the surface runoff source might have merged multiple point sources^27^. It is interesting to note that the PFOS decreased in both concentration and proportion could be explained by its almost absence from precipitation and runoff water.

Few previous studies have reported the occurrence of F-53B. This compound was mainly used as mist suppressant by chrome plating industry and it is also demanded as a PFOS alternative in China^36^. In this study, F-53B was detected in all sampling sites. It was unevenly distributed; Q-3 and W(M)-8 were found to be the most polluted sites in HWP and LWP, with concentrations of 29.42 (ng/L) and 8.14 (ng/L), respectively. This might be associated with nearby point source (Fig. 1). During LWP, Liangshui River was the most contaminated tributaries, whereas, in the HWP, there was no evident disparity in contamination status. Moreover, compared with PFOS, the observed concentrations of F-53B also dropped dramatically in HWP, whereas, the proportion remained at the same level, suggesting the PFCs compositional differences in runoff. (More information concerning F-53B was listed in the Supplementary Information).

### Source apportionment

In this part, three statistical approaches were employed to analyze the contributions of possible sources to PFC pollution in North Canal Catchment.

### Source Categories

PCA and HM-HCA were applied to aggregating the distributing pattern of PFCs in order to investigate the possible sources categories. These methods have been proved effective and widely used in source apportionment of environmental pollutants^9^,^30^,^40^.

The results of PCA (Fig. 3(A,B)) states that in both two samplings, concentrations of PFCAs (PFBA, PFPeA, PFHxA, PFHpA and PFNA (the clustered group)) were correlated, while PFOS, PFBS or F-53B were independent from other compounds. According to the reported source marker^4^,^23^, this environmental occurrence pattern should be associated with the source categories. The clustered compounds were widely used in industrial processes, however, due to the low concentration, and extensive and similar distribution patterns, they were more likely derived from nonpoint inputs. This is consisted with the previous studies that consumer products (e.g. textile, paper and food/pharmaceutical package) were the major PFCAs sources^4^,^29^,^30^. Thus, PC1 in both periods could be identified as the household emission source. PFOS and F-53B containing chemicals were used in galvanizing, nickel, chromium plating and anodizing industry^36^,^41^,^42^, therefore, PC 2 can be pinpointed as relevant industry^9^. This was proved by previous researches that highly concentrated PFOS and other PFSAs were observed in the effluents of metal industries impacted WWTP^4^,^30^,^43^. Moreover, although PC 3 in both periods was of minor importance, the distribution pattern of indicative compounds reflected the influence of certain sources. PFBS was used in aqueous film-forming foam (AFFF) to replace PFOS^41^ and it was also observed in the impacted surface water^29^,^44^,^45^, besides, PFBS in this study also presented in similar pattern (e.g. W5–7, downstream of the Airport). Accordingly, PC 3 can be identified as AFFF using units (e.g. airport and fire station). It is interesting to note that PFOA was not in the same group in HWP and LWP. This indicates that PFOA had sources other than domestic input, which were most likely to be food-packaging and paper grease-proofing treatments (Fig. 1)^30^.

### Geographic distribution of Sources

In cluster analysis, surface water contamination with similar source categories would be included in one group and the major pollutants would be displayed on the heat map^9^,^30^. As shown in Fig. 3(C,D), in both periods, the midstream of North Canal was clustered with Qinghe River and PFBS was the main chemical in the region, which was consisted with the geographic distribution of an airport and several fire station with large AFFF consumption (Fig. 1). In HWP (Fig. 3(C)), downstream of North Canal was grouped with all the tributaries but Qinghe River, which was otherwise clustered with the mid- and upstream of North canal. Besides, Compared with situations in LWP, a wider extension and jointed area was clustered as groups, and on the heat map, red color was less concentrated. This is another evidence to prove the mixing and diluting effect of runoff. In LWP (Fig. 3(D)), PFOS contamination was scattered and it was highlighted on the heat map. As expected, the result conformed to the geographic distribution of relevant industries, indicating surface water was heavily influenced by point emissions.

According to Beijing Statistical Yearbook^46^, 150,713 ton wastewater was generated in 2014 and entered natural surface water with or without proper treatment, and over 90% of which was discharged into North Canal. Besides, there were 3, 686 industrial enterprises above designed size (the main business income >RMB 20,000,000), among which over 40% were PFCs related industries and together they account for 17% total industrial output value (Supplementary Table S8).

### Impact of nonpoint source

In several previous studies, runoff water has been regarded as an important source of PFCs in surface water, and it was reported that precipitation contributed over 50% of the overall loads in some areas^33^,^47^. Nevertheless, these studies only considered the first flush of precipitation, which probably only caused minor runoff with very high pollutant loadings, but the effects of consecutive rainfall and large runoff were not taken into consideration. North Canal catchment is the largest drainage receptor of Beijing, therefore, its contamination status is heavily influenced by precipitation. In this study, we sampled the surface water right after consecutive storms. Figure 2 clearly reveals that PFCs concentrations in LWP were higher than those in HWP in almost all sampling sites. This circumstance indicates that the continuous rainfalls will bring rainwater with lower pollutant loading into the rivers, compared with the reported highly contaminated first flush, and hence, they played a dilution role^27^.

**Figure 4:**
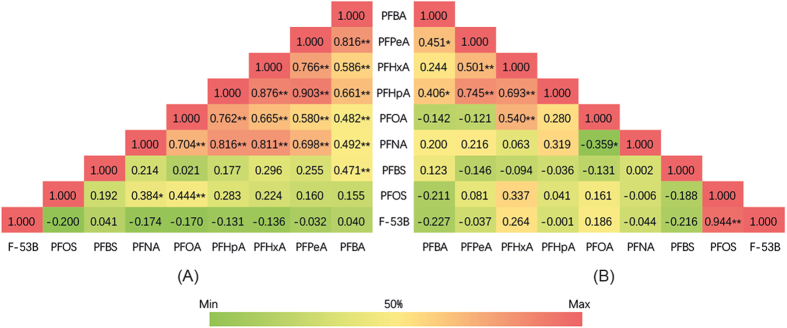
Pearson correlation analysis of PFCs. (**A**) HWP; (**B**) LWP; *indicates statistically significant at the level of p < 0.05; **indicates statistically significant at the level of p < 0.01.

In order to discriminate the impact of runoff, the correlation among each compound was studied. Pearson correlation coefficients between individual PFCs are displayed in Fig. 4. PFBA, PFPeA, PFHxA, PFHpA and PFNA are more related to each other in HWP (Fig. 4(a)) than in LWP (Fig. 4(b)). The results are consistent with the outcomes of HM-HCA and PCA. These compounds are often found in the domestic discharges^4^,^23^,^30^, and also highly influenced by traffic^34^ and deposition of atmospherically transformed telomer alcohols^48^, and therefore, they are usually concentrated in runoff^49^,^50^. Therefore, in this case, these compounds are probably brought into surface water by runoff, and the distribution pattern variation between two periods may also be explained by elevated runoff input^4^.

Concentration ratios can be employed to identify sources of pollutants, because of their different composition and behaviors in various environments^27^,^51^,^52^,^53^. In this case, precipitation runoff is considered as an extra source in HWP, leading to strong dilution of PFOS. Therefore, two indicators are proposed as tracers to discriminate the impact of runoff on HWP’s water environments: F-53B/PFOS concentration ratio and (PFBA + PFPeA + PFHxA + PFHpA)/PFOS concentration ratio. Firstly, F-53B and PFOS have the same number of fluorinated carbons and they are derived from similar point sources^35^,^36^. Also, Fig. 4 revealed that F-53B have a very close correlation with PFOS (P < 0.01) in LWP samples, rather than in HWP. Such contrast indicates source change, and consequently, the concentrations ratio can possibly be used as a source tracer. Secondly, PFBA, PFPeA, PFHxA and PFHpA share close statistical correlations with each other in all the samples (Fig. 4). Besides, as mentioned before, they were found highly loaded in nonpoint source, which varied much from the emission regularity of the point-source compound (i.e. PFOS). Therefore, another PFCs source indicator can be drew from (PFBA + PFPeA + PFHxA + PFHpA)/PFOS concentration ratio.

**Figure 5:**
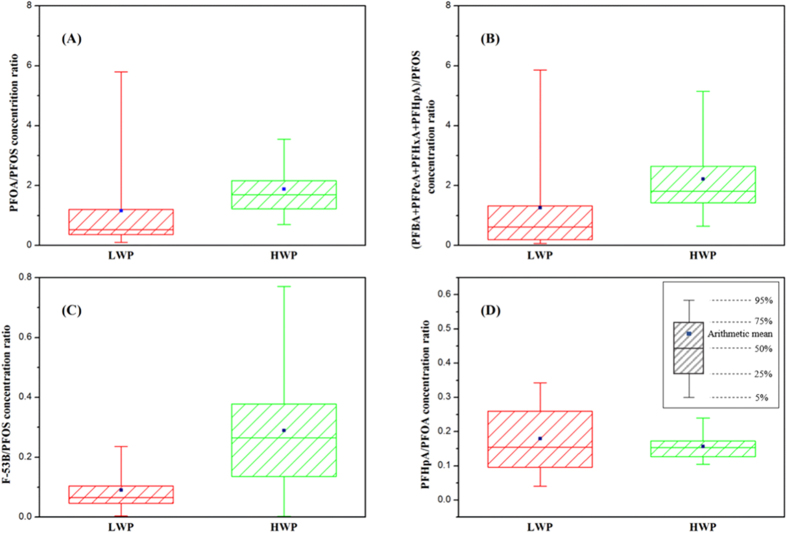
Boxplot of concentration ratios. (**A**) F-53B/PFOS; (**B**) PFHpA/PFOA; (**C**) (PFBA + PFPeA + PFHxA + PFHpA)/PFOS; (**D**) PFOS/PFOA.

F-53B/PFOS ratio in LWP ranged from 0.05 to 0.10 (25% percentile to 75% percentile, the same for other), and from 0.15 to 0.41 in HWP (Fig. 5(C)). (PFBA + PFPeA + PFHxA + PFHpA)/PFOS ratio ranged from 0.21 to 1.13 and 1.41 to 2.61, respectively (Fig. 5(B)). Given the disparity of pollution status and lack of enough validation, these measure might not be specific enough to absolutely identify nonpoint source. Nevertheless, clear difference has been shown and these ratios are potentially useful for the evaluation of the contributions from point source and non-point source to PFCs in multiple water environments. Thus further investigations of the various sources are needed to validate the proposed indicators.

PFOA/PFOS was reported typically greater than 1.1 in the surface water^27^. Nevertheless, this ratio is not suitable for the studied area, especially in dry weather (median: 0.53 for LWP), probably because China is the only one where PFOS is still being manufactured. Conversely, it is interesting to note that this ratio was significantly elevated to 1.70 (median) in HWP (Fig. 5(A)), and the rational explanation is the input from runoff. This theory is supported by several previous studies. The ratio in runoff is reported around 9, which could make contribution to the increment of this ratio in HWP^35^; a study conducted in Tianjin, a metropolis adjacent to Beijing, states that the frequency and content of PFOS is much less than most PFCAs, throughout all the seasons^54^.

Besides runoff, previous studies have also mentioned several other weighty nonpoint sources for surface water^6^,^27^,^35^,^49^,^55^. The ratio of PFHpA/PFOA (Fig. 5(D)) is regarded as an effective tracer of atmospheric deposition^9^,^41^,^56^. In this study, this ratio ranged from 0.03 to 0.34 with little variance between two sampling samplings, indicating the distribution of PFCs was not much affected by atmospheric deposition. Also, this ratio is used to measure the nearness to urbanized and industrialized areas^33^, which is consisted with the case in this study. PFCs precursors existed in wastewater, and they can be degraded/biodegraded into PFCs as they enter surface water^30^,^35^,^56^. PFCs loads had even been found increasing during wastewater treatment process^43^. It is reported that biodegradation of the same precursors produced mainly PFOA and secondarily PFNA^35^. In the present study, the ratio of PFNA/PFOA ranged from 0 to 0.18 for both two samplings. PFNA and PFOA had close correlations in HWP (p < 0.01) and LWP (p < 0.05), indicating the possibility that precursors degradation is another source.

## Conclusion

This study is the first large-scale surface water investigation of PFCs and alternative F-53B occurrence in Beijing, the capital of China. It provides important data on the occurrence and distribution patterns of PFCs in different water periods. In North Canal basin, Liangshui River is the most polluted tributary in both high-water period and low-water period. The rivers flowing through the central city tends to show higher loadings than those running past rural area. The PFCs contamination of surface water is found to be dominated by PFOS, PFOA and PFBS all the time, which are considered to come mainly from relevant industrial discharges. In HWP, large amount of runoff mixes and dilutes PFCs concentration in surface water and it is identified as an input, because it obviously changes the PFCs compositional profile between different water periods. Moreover, source apportionment was achieved by investigating multivariate statistical techniques to distinguish the traits of pollution and determine the sources. The consequences are geographically consisted with known sources. Two new composition ratios are proposed as indicators to discriminate the influence nonpoint source. F-53B/PFOS <0.07 and (PFBA + PFPeA + PFHxA + PFHpA)/PFOS <0.62 indicate the impact of point source. But nonpoint source, specially runoff, comes to make a difference when the ratios >0.28 and >1.87, respectively. The application of the methodology combined with PCA, HM-HCA, and correlation analysis have greatly reduced the complexity of the data, allowing to build up meaningful relation between sources and pollution status. This technique is believed to make a contribution to optimizing river/environment monitoring system.

To the best of our knowledge, this is the first systematic report of F-53B together with various PFCs in a regional environment in China, which was found heavily used in this region and its occurrence and distribution patterns varied much along seasonal change. Its occurrence is similar to the level of PFPeA, PFHxA, PFHpA and PFNA. In the LWP, F-53B have the same source category with PFOS; whereas, it can be brought into surface water by runoff in HWP, which is not considered as a major source for PFOS. F-53B was first synthesized in China and had been produced for more than 30 years; nevertheless, few studies had attached importance to this compound. Recently, as a part of the performance of Stockholm Convention, Chinese ministry of environmental protection have been encouraging the application of PFOS’s alternatives (including F-53B)^57^, so the larger usage and output of F-53B can be expected. Besides, owing to the lacking knowledge of its toxicity, fate and occurrence in various environments, sustained attention should be paid to the environmental impacts of its large output and usage.

## Materials and Methods

### Standards, Reagents and other materials

In this study, 11 PFCs were selected for target compounds, including 7 PFCAs (4A to 8A) and 4 PFSAs (4S, 6S, 8S and F-53B) (detailed in Supplementary Information). ^13^C_4_-PFBA, ^13^C_5_-PFPeA, ^13^C_2_-PFHxA, ^13^C_4_-PFHpA, ^13^C_4_-PFOA, ^13^C_5_-PFNA, ^13^C_2_-PFDA, ^13^C_4_-PFBS, ^13^C_3_-PFHxS(Na), ^13^C_4_-PFOS(Na) and ^13^C_8_-PFOS(Na) were purchased from Wellington Laboratories Inc. (ON, Canada) with purities of >98% as internal standards. F-53B was obtained from Shanghai Synica Co., Ltd. (Shanghai, China). Solvents for cleaning and extraction purposes were supplied by J.T. Baker (NJ, USA) and ultrapure water was used (18 MΩ·cm, Millipore, MA).

### Sampling

Water samples were collected from North Canal Basin, which is located in the northeast Beijing with a catchment area of 4,293 km^2^. This is one of the most urbanized and industrialized area in the world, resided by 70% population and receiving 90% city drainage of Beijing^58^,^59^. As shown in Fig. 1, all the major WWTPs (Supplementary Table S9) in Beijing were located near the tributaries of North Canal, which bring in more than 3 million tons of wastewater per day^59^. Due to the insufficiency of wastewater collection and treatment system, the catchment was considered as the most polluted water environment in Beijing (Supplementary Table S1).

Total 34 Samples were collected along main stream (13 sites) and 6 tributaries (21 sites) in Beijing section of North Canal within one day (July and December of 2015 respectively, Fig. 1 and Table S1). The high-water period (HWP) in Beijing usually lasts from June to September, and the low-water period (LWP) lasts from October to March. According to Beijing water authority^60^, before the first sampling, several intense storms (detailed in Supplementary Information) hit Beijing city and caused major runoff. While during the second sampling, there was very little rain in Beijing. Therefore, runoff amount and flow rate were dramatically different between two samplings.

Water samples were taken 0.5 m under the surface and stored in 500 mL polypropylene (PP) bottles with screw caps (Vitlab, Grossostheim, Germany). PP is the most suitable material for anionic PFCs^61^. All samples were carried back to laboratory within 12 hours when they were stored in a refrigerator before further actions.

### Sample pretreatment

Samples were filtered under vacuum through glass fiber filters (GF/F. 47 mm, Whatman, Kent, UK) to remove the suspended particulate matters (SPM). After filtration, PFCs samples were extracted and fractionated respectively. 25 ng internal standard was added and aged for 30 minutes. The PWAX-SPE cartridge (150 mg, 6 ml, AGELA Technologies, Tianjin, China) was conditioned by passing 4 mL 0.5% ammonia methanol solution, 4 mL methanol and 4 mL high purity water (HPW), respectively. The analytical sample was loaded onto the cartridge, and the aqueous eluate was collected at a rate of 3–5 mL/min. The SPE cartridge was then washed by 4 mL 25 mM sodium acetate solution, and dehydrated by pump air at 10 bar pressure. The analytes were eluted with 4 mL methanol and 4 mL 0.5% ammonia methanol solution successively. The elutes were concentrated by a stream of purified N_2_ to 250 μL. Prior to instrumental analysis, 250 μL ultrapure water and 25 ng injection standard, was added to each sample. Samples were finally filtered by 0.22 um microfiltration membrane (Pall Inc., Shanghai, China) and prepared to be analyzed.

### HPLC-MS/MS analysis

Instrumental analysis was performed by a high-performance liquid chromatograph interfaced with a tandem mass spectrometer (HPLC-MS/MS) operated in electrospray negative ionization mode. Target compounds were separated on a Xbrige BEH C18 column (3.5 μm, 3.0 × 150 mm, Waters lnc., Shanghai, China) using an UltiMate 3000 HPLC (Dionex by Thermo Fisher Scientific Inc., Massachusetts, USA) and detected by an API 3200 triple quadrupole mass spectrometer (AB SCIEX, ON, Canada). The mobile phase flowed at a rate of 0.3 mL/min, consisting of 2 mM ammonium acetate (A) and methanol (B). The elution program was started with 40% B, and increased to 100% B from 1 min to 7 min. After another 3.5 min, it returned to 40% B and kept for 3 min. Target compounds were measured by MS/MS in multiple reaction monitoring (MRM) mode. Ion transitions monitored were, m/z 498.8 > 98.9(80) for PFOS, m/z 412.8 > 369.0 for PFOA, 502.7 > 98.9 for ^13^C_4_-PFOS and 416.7 > 269.9 for ^13^C_4_-PFOA (other substances were discussed in Supplementary Information).

### Statistical analysis and data processing

In this study, statistical analyses were carried out by using IBM SPSS Statistics 21 (IBM Lnc., New York, USA), Origin 8 (OriginLab Corp., Massachusetts, USA) and R (the R foundation., Vienna, Austria). Values below detection limits were assigned as one-half of the limits, prior to statistical analysis. Two compounds (PFHxS, PFDA) were not quantifiable at all the sites. Therefore, they were not taken into further discussion or the following sections. More details were given in the Supplementary Information.

### QA/QC

A strict quality control regime was operated during the experiment. Field blanks, transport blanks and solvent blanks were conducted with every sample set. PFC quantification was based on the calibration curves of which the correlation coefficients were all higher than 0.99. The limit of detection (LOD) and limit of quantification (LOQ) were defined as the peak of analyte that yield a signal-to-noise (S/N) ratio of 3:1 and 10:1, respectively. The procedure recoveries ranged from 83% to 133% at moderate concentration (5 ng standard added), and 62% to 126% at high concentration (20 ng). Detailed QA/QC measurements of PFCs in water samples were given in Supplementary Information.

## Additional Information

**How to cite this article**: Zhang, Y.-Z. *et al.* Occurrence and source apportionment of Per- and poly-fluorinated compounds (PFCs) in North Canal Basin, Beijing. *Sci. Rep.* **6**, 36683; doi: 10.1038/srep36683 (2016).

**Publisher’s note**: Springer Nature remains neutral with regard to jurisdictional claims in published maps and institutional affiliations.

## Supplementary Material

Supplementary Information
