# Supplementary material for: Occurrence and source apportionment of Per- and poly-fluorinated compounds (PFCs) in North Canal Basin, Beijing
Source: Sci Rep. 2016 Nov 15;6:36683. doi: 10.1038/srep36683 (PMC5109260; doi:10.1038/srep36683)
Supplement: Supplementary Information [file srep36683-s1.pdf]

Supplementary Information

Occurrence and source apportionment of Per- and poly-fluorinated compounds (PFCs) in North Canal Basin, Beijing

Yi-Zhe Zhang [1], Bin Wang [1] *, Wei Wang [2], Wen-Chao Li [2], Jun Huang [1], Shu-Bo Deng [1], Yu-Jue Wang [1], Gang Yu [1]

([1] Beijing Key Laboratory of Emerging Organic Contaminants Control, State Key Joint Laboratory of Environmental Simulation and Pollution Control, Collaborative Innovation Center for Regional Environmental Quality, School of Environment, Tsinghua University, Beijing, 100084, China, [2] CSD IDEA (Beijing) Environmental Test&Analysis Co., Ltd., Beijing 100192, China. *E-mail: thuwb@tsinghua.edu.cn)

Content

1. **Study area**
2. **Precipitations during two samplings**
3. **Production and usage of PFCs in China**
4. **General information of F-53B**
5. **Heat map-hierarchical cluster analysis (HM-HCA)**
6. **Principal component analysis (PCA)**
7. **Environmental risk assesment**
8. **Tables**

    Table S1 Description of sampling locations

    Table S2 PFCs measured in this study with QA/QC information including monitoring transitions, procedure recovery (PR), limit of detection (LOD), limit of quantification (LOQ), declustering potential (DP), and collision energy (CE) (Mean: Arithmetic mean; RSD: Relative Standard Deviation; n indicates the number of samples analyzed.)

    Table S3 HPLC conditions (a); MS conditions (b)

    Table S4 PFCs concentrations from high-water period (ng/L) in North Canal and its tributaries

    Table S5 PFCs concentrations of low-water period (ng/L) in North Canal and its tributaries

    Table S6 PFCs concentrations in water samples from different studies

    Table S7 Component Score Coefficient Matrix of Principal component analysis. (a) HWP; (b) LWP.

    Table S8 Survey of Beijing industrial enterprises and PFCs related enterprises (2015)

    Table S9 WWTPs with a treatment capability of over 80,000 m3/d in North Canal Basin

9. **Figures**

    Fig. S1 the average concentration of PFCs in different water periods

    Fig. S2 Proportions of the average concentration of PFCs in North Canal Basin

1. Study area

North Canal catchment is one of the four north tributaries of the Haihe River. It is situated in the northeast Beijing with a catchment area of 4,293km^2 and a population of more than 13 million. This region takes up just 27% of the urban area, but is resided by more than 70% of population and possesses around 80% of GDP of Beijing. The watershed covers the most industrialized and urbanized area of Beijing. Therefore, Due to the large amount of discharges (more than 90% sewage of Beijing city) and imperfect environmental management, North Canal Basin is recognized as one the most contaminated water bodies throughout Beijing. Recently, with the industrial restructuring and transfer, the water quality has been reported much improved. Nevertheless, there still exists many PFCs related facilities, and north canal basin is still subjected to PFCs pollution to quite serious degree.

2. Precipitations during two samplings

According to Beijing Water Authority[1], a week before HWP sampling , the average daily accumulating rainfall (DAR) in Beijing reached 55mm. Continuous rainfall with 4 major storms hit Beijing city. Compared with HWP, average DAR during the week before LWP sampling was only 3 mm. Besides, the precipitation was unevenly distributed. North Canal basin was a hard-hit area. Haidian district, for example, experienced 4 heavy storms with 24h average precipitation of 19.6 mm, 12.6 mm, 59.3 mm and 64.8 mm (Beijing Haidian District water Authority, 2015), before the sampling day.

3. The occurrence, production and usage information of PFCs in China

Perfluorooctane sulfonate (PFOS) and Perfluorooctanoic acid (PFOA) are the most often applied and detected perfluorinated compounds in history. As we can see from Table S6, pollution status of these two chemicals in Beijing region, are in the moderate level all around the world. However, it is noteworthy that

the level of PFBS, an important PFOS alternative, exceeds most other places.

In China, from 2002 to 2008, about 100 tons of PFOS is produced each year. Perfluorootane sulfonates (PFOS) are mainly used for metal plating, aqueous fire-fighting foams (AFFFs) and sulfluramidin China, and the use amount is about 30-40 t/y, 25-35 t/y and 4-8 t/y respectively[2]. For PFOA, the production volume is at the same level as PFOS, and it is mainly applied to chemical production, food processing and paper treatment industries[3].

4. General information of F-53B

Perfluorooctane sulfonic acid (PFOS) was long used as a stain repellent. After being found ubiquitously in human as well as the environment, it was voluntarily phased out by its main producer 3M and replaced in many applications with perfluorinated compounds, i.e., perfluorononanoic acid (PFNA). In 2009, PFOS and its derivatives usage was generally restricted under the Stockholm Convention on Persistent Organic Pollutants.

The trade name of potassium 2-(6-chloro-1,1,2,2,3,3,4,4,5,5,6,6-dodecafluor-ohexyloxy)-1,1,2,2-tetrafluoroethanesulfonate (6:2 Cl-PFAES) is F-53B ($C_8ClF_{16}O_4SK$). [4]) reported that this chemical was largely used as mist suppressants in electroplating industry to replace perfluorooctane sulfonate acid and its salts (PFOS, $C_8F_{17}SO_3^-$). F-53 was first developed as a mist suppressant for the hard chrome plating industry, by the Shanghai Institute of Organic Chemistry, Chinese Academy of Sciences in 1975. After successful demonstrations in four local electroplating plants in Shanghai, F-53 was found to be excellent in performance but high in synthesis cost. F-53B is the modified version of F-53, with the replacement of one fluorine atom by a chlorine (Fig. SI-1). This modification was made to simplify the production process,

reduce the cost where chlorination is used in the last step, and prevent the use of toxic and expensive chemicals (e.g., $SbCl_5$ and SbF_3). Therefore, the commercialized product was F-53B instead of F-53.

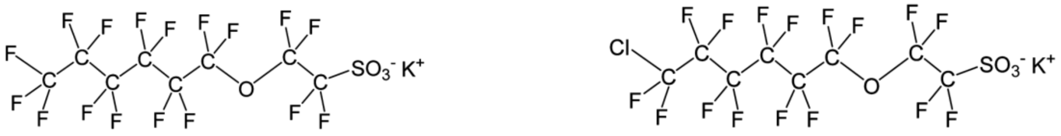

(a) (b)

Fig. SI-1 (a) chemical structure of F-53; (b) chemical structure of F-53B

5. Heat map-hierarchical cluster analysis (HM-HCA)

Cluster analysis, as a multivariate statistical method, has been largely used in source apportionment of organic pollutants. In the present work, cluster analysis serves to aggregate the PFCs patterns of surface water samples into clusters in such a way that the patterns in any one cluster are similar to each other as possible within one cluster, and as different from each other as possible in comparison to other clusters. During cluster analysis, the closest pair of PFCs samples was linked into a cluster according to a similarity measure. Values blow limits were assigned a value of one-half of the limits. The concentration of each of the five PFCs was normalized by the total concentration of PFCs in every setting. The squared Euclidean distance was used to measure similarity among monitoring points. The clustering was carried out by minimizing the sum of squared Euclidean distance. More information are available from [5]). The cluster and correlation analysis were performed using R (the R foundation, Welthandelsplatz, Vienna).

6. Principal component analysis (PCA)

PCA was introduced in this study to characterize the potential relationship of the distribution among 9

PFCs (including 31 water samples and 9 individual PFCs compounds for LWP and 34 water samples and 9 individual PFCs compounds for HWP) extracting three principle components through dimension reduction. Kaisex-Meyer-Olkin test was used to test if the partial correlation among variances was small and Bartlett's test of Sphericity was used to test if the variances were independent among each other. The variances in this study had passed all the test. The principle components were extracted using correlation matrix, while the rotation of the factors was set as varimax with Kaiser Normalization. Three components were extracted with the initial eigenvalues greater than 1.0 for both two periods. As displayed in Fig. 3, for HWP, component 1 (53.38% of the total variance) was highly loaded with PFPeA, PFHxA, PFHpA, PFOA, PFNA and PFBA; component 2 (14.56% of the total variance) mainly contributed by PFOS; component 3 (11.42% of the total information) possessed high level of PFBS. For LWP, three components take up 30.69%, 25.71% and 15.35%, respectively and mainly correspond to PC1 (PFPeA, PFHpA, PFHxA PFBA and PFNA), PC 2 (PFOS and F53B), and PC3 (PFOA), in the same order. Results of the scree test indicated that the eigenvalue of the first three components located in the steep polyline before the elbow appeared in the scree plot. The first three components explained over 79% of the cumulative variance with each component contributing at least 11%. The results of the PCA can meet the demand for further analysis. The principal component analysis was performed by IBM SPSS Statistics 21 (IBM Lnc. Armonk, New York).

7. Environmental risk assessment

In this study, environemtal risk assessment was carried out according to previously reported hazard quotient (HQ) method[6]. The most sensitive predicted no effect concentrations (PNECs) are selected, and the data acquired form this study are used as MECs. According to Table SI-1, generally, the PFCs pollution status may not pose environmental risks, however, further attention should be paid to those compounds.

Table SI-1 Environment risk assessment of 9 PFCs, predicted no effect concentration (PNEC), measured environemtal concentration (MEC), Hazard quotient (HQ), high-water period (H), low-water period (L).

Compound	$PNEC_{aquatic}$ (μg/L)	Reference	Water period	MEC (ng/L)	HQ ($\times 10^{-6}$)
PFBA	-		H	0.04-4.70	-
			L	0.00-23.80	-
PFPeA	32	7	H	0.36-2.58	11.25-80.63
			L	0.00-7.88	0-246.25
PFHxA	97	7	H	0.45-1.92	4.64-19.79
			L	0.00-7.76	0-80.00
PFHpA	-		H	0.22-1.38	-
			L	0.80-3.70	-
PFOA	1250	8	H	1.61-5.44	1.29-4.35
			L	4.21-98.52	3.37-78.82
PFNA	100	7	H	0.09-0.54	0.90-5.40
			L	0.00-1.79	0-17.90
PFBS	-		H	0.30-5.36	-
			L	0.00-77.00	-
PFOS	25	8,9	H	0.25-5.40	10.00-216.00
			L	2.24-121.62	89.60-4864.80
F-53B	-		H	0.00-29.42	-
			L	0.03-8.14	-

Table S1 Description of sampling locations

River		Site	Latitude	Longitude	Ambient description
Main stream	Upper reach (Wenyu River)	W(U)-1	40°07.965′	116°20.343′	Green land
		W(U)-2	40°09.080′	116°24.689′	Green land
		W(U)-3	40°08.854′	116°26.647′	Green land
		W(U)-4	40°06.903′	116°29.378′	Green land
	Middle reach (Wenyu River)	W(M)-5	40°03.401′	116°32.666′	Green land
		W(M)-6	40°01.837′	116°34.304′	Green land
		W(M)-7	39°59.900′	116°38.431′	Green land
		W(M)-8	39°59.214′	116°38.507′	Green land
	Down reach (North Canal)	W(D)-9	39°54.569′	116°40.440′	Residential area
		W(D)-10	39°53.314′	116°43.632′	Factories around
		W(D)-11	39°48.877′	116°46.553′	Green land
		W(D)-12	39°47.560′	116°47.199′	Green land
		W(D)-13	39°45.989′	116°52.675′	Green land
Qinghe River		Q-1	40°01.143′	116°19.034′	Heavy traffic area
		Q-2	40°04.870′	116°28.898′	Green land
		Q-3	40°03.404′	116°25.401′	Heavy traffic area, factories around
		Q-4	40°04.870′	116°28.898′	Green land
Bahe River		B-1	39°58.437′	116°31.912′	Residential area
		B-2	39°58.149′	116°33.463′	Residential area
		B-3	39°58.046′	116°35.532′	Residential area
		B-4	39°57.398′	116°37.663′	Factories around
Tonghui River		T-1	39°54.402′	116°31.414′	Residential area
		T-2	39°54.313′	116°33.219′	Heavy traffic area
		T-3	39°54.505′	116°34.247′	Residential aera, heavy traffic area
		T-4	39°54.637′	116°39.054′	Residential aera, heavy traffic area
Lianghsui River		L-1	39°47.835′	116°27.688′	Residential area
		L-2	39°45.718′	116°32.284′	Residential area
		L-3	39°47.501′	116°38.465′	Factories around
		L-4	39°50.596′	116°41.933′	Residential area
		L-5	39°48.257′	116°45.795′	Factories around
Xiaozhong River		A-1	39°56′20.71″	116°39′31.26″	Residential area
		A-2	39°59′7.28″	116°40′7.76″	Factories around
Yunchaojian River		C-1	39°55′18.21″	116°40′53.36″	Heavy traffic area
		C-2	39°54′57.19″	116°43′46.84″	Heavy traffic area

The symbols in parentheses are omitted in some figures

This table is the description of Fig. 1

Table S2 PFCs measured in this study with QA/QC information including monitoring transitions, procedure recovery (PR), limit of detection (LOD), limit of quantification (LOQ), declustering potential (DP), and collision energy (CE) (Mean: Arithmetic mean; RSD: Relative Standard Deviation; n indicates the number of samples analyzed.)

Compound	Abbreviation	MS/MS transition	PR (n=5, 5ng)	PR (n=5, 20ng)	LOD	LOQ	DP	CE
			% Mean ±RSD	% Mean ±RSD	(ng/L)	(ng/L)	(-V)	(-eV)
**Target analytes**	**PFCAs**							
Perfluorobutanoic acid	PFBA	212.6 → 168.9	88±16	109±13	0.020	0.078	15	8
perfluoropentanoic acid	PFPeA	262.7 → 218.9	84±6	108±9	0.009	0.038	15	6
perfluorohexanoic acid	PFHxA	312.7 → 268.9	106±6	89±13	0.007	0.027	15	8
perfluoroheptanoic acid	PFHpA	362.7 → 318.9	105±7	76±6	0.006	0.025	15	10
perfluorooctanoic acid	PFOA	412.8 → 369.0	118±16	81±16	0.003	0.013	20	10
perfluorononanoic acid	PFNA	462.8 → 418.9	83±8	62±36	0.003	0.010	15	12
perfluorodecanoic acid	PFDA	512.8 → 468.9	89±24	74±5	0.002	0.006	15	14
	**PFSAs**							
Perfluorobutane sulfonate	PFBS	298.7 → 80.0	101±14	108±14	0.002	0.009	55	50
Perfluorohexane sulfonate	PFHxS	398.7 → 80.0	89±9	92±18	0.001	0.005	65	70
Perfluorooctane sulfonate	PFOS	498.8 → 80.0	93±9	94±18	0.003	0.011	85	88
Perfluoroalkyl ether potassium sulfonate	F-53B	530.9→ 351.0	106±11	100±9	0.001	0.003	9	26
**Internal standard**								
Perfluoro-*n*-[$^{13}C_4$] butanoic acid	$^{13}C_4$-PFBA	216.7 → 171.9	-	-	-	-	15	8
perfluoro-*n*-[$^{13}C_5$] pentanoic acid	$^{13}C_5$-PFPeA	267.7 → 223.1	-	-	-	-	40	18
Perfluoro-*n*-[$^{13}C_2$] hexanoic acid	$^{13}C_2$-PFHxA	314.7 → 269.9	-	-	-	-	15	10

perfluoro-*n*-[$^{13}C_4$] heptanoic acid	$^{13}C_4$-PFHpA	366.6 → 322.0	-	-	-	-	15	14
Perfluoro-*n*-[$^{13}C_4$] octanoic acid	$^{13}C_4$-PFOA	416.7 → 371.9	-	-	-	-	20	10
Perfluoro-*n*-[$^{13}C_5$] nonannoic acid	$^{13}C_5$-PFNA	467.8 → 422.9	-	-	-	-	20	12
Perfluoro-*n*-[$^{13}C_2$] decanoic acid	$^{13}C_2$-PFDA	514.6→ 469.8	-	-	-	-	20	12
Perfluoro-*n*-hexane [$^{18}C_3$] sulfonate	$^{18}C_2$-PFHxS	401.7 → 80.0	-	-	-	-	75	70
Perfluoro-*n*- octane [$^{13}C_4$] sulfonate	$^{13}C_4$-PFOS	502.7 → 80.0	-	-	-	-	80	98
**Syringe standard**								
Perfluoro-*n*- octane [$^{13}C_8$] sulfonate	$^{13}C_8$-PFOS	507.0 → 80.0	-	-	-	-	45	50

Table S3 HPLC conditions (a); MS conditions (b)

(a)

Analytical column	Waters XBridge BEH C18 3.5μm, 3.0×150mm column	
Column temperature	30 ℃	
Injection volume	10 μL	
Mobile phase	A= 2 mM ammonium acetate<br>B= 100% Methanol	
Run time	10.5 min + 3 min post time	
Flow rate	0.3 mL/min	
Gradient	Time (min)	Mobile phase
	0	40% B
	7	100% B
	10.5	40% B

(b)

Acquisition parameters	Electronic Spray Ion (ESI) mode, negative ionization; multiple reaction monitoring (MRM)
Source gas temperature	450 ℃
Ionspray voltage (-)	4500 V

Table S4. PFCs concentrations at high-water period (ng/L) in North Canal and its tributaries, arithmetic average concentration (Ave), relative standard deviation (RSD)

HWP Site	PFCAs						PFSAs			ΣPFCs	Ave	RSD
	PFBA 4A	PFPeA 5A	PFHxA 6A	PFHpA 7A	PFOA 8A	PFNA 9A	PFBS 4S	PFOS 8S	F-53B 8S			
W(U)-1	1.12	0.78	1.39	0.86	5.44	0.48	3.31	3.16	0.46	16.98		
W(U)-2	0.04	0.67	0.94	0.42	2.29	0.35	2.73	2.06	0.07	9.53		
W(U)-3	2.02	0.78	1.10	0.55	3.19	0.41	4.85	1.78	0.36	15.04		
W(U)-4	0.09	0.74	0.91	0.56	3.13	0.39	2.62	5.40	0.02	13.86	13.85	20%
W(M)-5	2.21	0.64	0.81	0.32	2.38	0.21	5.36	2.56	0.49	14.99		
W(M)-6	0.94	0.52	0.85	0.35	2.18	0.20	4.54	1.66	0.04	11.28		
W(M)-7	2.30	0.61	0.72	0.37	2.36	0.23	4.84	2.21	0.51	14.15		
W(M)-8	0.82	0.43	0.64	0.25	1.95	0.17	2.42	1.34	0.18	8.20		
W(M)-9	1.66	0.55	0.77	0.36	2.30	0.20	4.00	2.04	0.84	12.72	12.27	20%
W(D)-10	1.37	0.74	1.84	0.71	4.10	0.31	2.21	1.90	0.54	13.72		
W(D)-11	1.09	0.65	0.79	0.40	3.24	0.24	2.10	1.63	0.74	10.86		
W(D)-12	2.42	1.36	1.10	0.79	3.28	0.35	2.98	3.27	0.39	15.92		
W(D)-13	2.11	1.49	1.19	0.72	3.66	0.35	2.45	1.90	0.78	14.64	13.78	14%
Q-1	1.67	0.82	0.71	0.38	2.46	0.16	4.72	1.11	0.42	12.46		
Q-2	1.53	0.82	0.64	0.33	2.21	0.10	4.54	1.26	0.29	11.73		
Q-3	1.94	0.77	0.69	0.32	2.11	0.18	3.73	0.95	29.42	40.12		
Q-4	2.21	0.69	0.64	0.29	2.71	0.14	3.84	3.22	1.39	15.13	19.86	59%
B-1	0.32	0.44	0.47	0.29	2.08	0.09	0.30	0.64	9.06	13.69		
B-2	1.74	0.56	0.60	0.38	3.57	0.18	1.37	2.25	0.76	11.41		
B-3	0.20	0.54	0.63	0.45	2.63	0.17	0.39	0.86	0.09	5.96		
B-4	1.54	0.56	0.61	0.37	2.71	0.26	1.83	1.58	0.60	10.05	10.28	27%
T-1	0.82	0.36	0.51	0.26	2.06	0.16	0.91	0.84	0.44	6.35		
T-2	0.84	0.49	0.56	0.40	2.32	0.14	1.18	1.61	1.24	8.78		
T-3	0.73	0.44	0.53	0.34	2.70	0.14	0.99	1.78	0.24	7.88		
T-4	1.08	0.47	0.65	0.34	2.79	0.15	1.34	3.97	1.11	11.91	8.73	23%
L-1	0.84	0.37	0.45	0.32	3.90	0.30	1.42	2.34	1.45	11.40		
L-2	1.36	0.66	0.73	0.41	3.56	0.24	1.39	2.80	2.16	13.32		
L-3	2.41	1.52	1.21	0.73	4.05	0.28	2.16	1.14	0.31	13.81		
L-4	4.21	2.58	1.92	1.38	4.11	0.54	4.35	1.96	0.74	21.78		
L-5	4.70	2.30	1.59	1.04	4.65	0.54	2.15	2.65	0.55	20.16	16.09	25%
A-1	0.13	0.41	0.65	0.22	1.75	0.27	0.81	0.25	0.00	4.49		
A-2	1.37	0.48	1.07	0.26	2.01	0.31	2.89	1.65	0.59	10.64	7.56	41%
C-1	0.37	0.45	0.62	0.30	1.61	0.13	0.96	0.59	0.10	5.13		
C-2	1.87	0.47	0.59	0.29	2.80	0.16	3.11	1.65	0.44	11.38	8.25	38%
Ave	1.47	0.77	0.86	0.46	2.89	0.25	2.61	1.94	1.67	12.92		

RSD	71%	66%	44%	55%	31%	48%	56%	53%	307%	48%

Table S5. PFCs concentrations at low-water period (ng/L) in North Canal and its tributaries, arithmetic average concentration (Ave), relative standard deviation (RSD)

LWP Site	PFCAs						PFSAs			ΣPFCs	Ave	RSD
	PFBA 4A	PFPeA 5A	PFHxA 6A	PFHpA 7A	PFOA 8A	PFNA 9A	PFBS 4S	PFOS 8S	F-53B 8S			
W(U)-1	6.22	0.00	4.02	1.76	8.64	1.13	3.94	61.40	5.12	92.22		
W(U)-2	0.00	0.00	3.46	2.04	12.30	0.97	4.14	16.52	1.64	41.07		
W(U)-3	0.00	0.00	4.20	2.04	13.24	0.45	3.96	29.00	2.04	54.93		
W(U)-4	0.00	0.00	4.96	2.38	28.52	1.17	3.58	80.40	3.72	124.73	78.24	42%
W(M)-5	4.60	0.00	2.02	1.57	4.59	0.45	34.20	10.72	0.04	58.19		
W(M)-6	4.66	0.00	2.24	1.59	7.03	0.45	53.80	4.22	1.00	74.99		
W(M)-7	0.00	0.00	4.28	1.67	18.42	0.00	36.60	17.76	1.57	80.31		
W(M)-8	0.00	0.00	5.08	2.22	54.92	0.00	22.20	115.00	8.14	207.56		
W(M)-9	6.38	0.00	3.84	1.22	14.64	0.45	3.86	81.40	4.50	116.29	107.47	50%
W(D)-10	23.80	0.00	2.60	1.64	13.55	0.00	4.54	2.24	1.27	49.65		
W(D)-11	6.04	0.00	4.12	1.84	33.12	0.00	4.94	65.80	4.28	120.14		
W(D)-12	11.96	6.12	4.40	3.42	11.33	0.91	7.62	9.44	0.26	55.47		
W(D)-13	6.52	4.38	3.90	2.04	9.33	0.45	5.32	11.02	0.09	43.05	67.08	46%
Q-3	17.34	0.00	3.94	2.74	9.81	1.79	56.20	4.10	0.43	96.35		
Q-4	6.30	0.00	2.14	1.41	5.43	0.45	77.00	10.56	0.10	103.40	99.88	4%
B-1	5.60	3.24	2.50	1.95	34.31	0.00	4.50	10.02	0.33	62.46		
B-2	4.68	1.81	2.41	2.53	14.66	1.71	2.11	16.38	0.77	47.07		
B-3	3.66	0.00	1.90	1.15	9.77	0.45	2.40	11.06	0.03	30.42		
B-4	5.10	0.00	3.00	1.33	13.90	0.94	2.84	6.00	0.82	33.93	43.47	29%
T-1	4.50	0.00	2.94	1.30	12.30	1.09	1.95	14.12	1.83	40.02		
T-2	4.95	0.00	2.58	1.41	6.60	0.69	2.57	16.71	1.76	37.26		
T-3	4.70	0.00	2.84	1.70	17.72	0.96	1.88	12.62	1.75	44.16		
T-4	4.90	0.00	7.76	3.08	98.52	0.00	3.92	17.00	1.39	136.57	64.50	65%
L-1	4.10	0.00	2.32	1.11	9.53	0.45	2.80	45.42	2.11	67.84		
L-3	12.94	7.88	6.38	3.70	11.73	1.35	7.86	110.82	6.13	168.79		
L-4	13.80	6.06	6.54	3.20	10.55	1.03	9.98	34.22	2.15	87.53		
L-5	11.54	6.92	6.04	3.40	6.69	0.45	6.92	18.42	0.92	61.31	96.37	45%
A-1	0.00	0.00	0.00	2.22	6.90	0.45	0.00	13.72	2.82	26.11		
A-2	0.00	0.00	2.56	0.80	4.21	0.45	4.30	121.62	7.49	141.43	83.77	69%
C-1	2.83	0.00	2.62	1.28	8.62	0.45	6.29	16.23	1.17	39.49		
C-2	5.76	0.00	2.64	1.59	10.41	0.45	7.60	13.38	0.74	42.57	41.03	4%
Ave	5.90	1.17	3.56	1.98	16.82	0.63	12.57	32.17	2.14	76.95		
RSD	93.09%	205.25%	45.57%	38.25%	109.85%	77.26%	150.12%	109.11%	101.21%	57.85%		

Table S6 Comparion of PFCs concentrations in water samples with other studies

Location	Concentration (ng/L)			Reference
	PFOS	PFOA	PFBS	
North Canal, China	0.25-5.40 (1.94) [1]<br>2.24-121.62 (32.17) [2]	1.61-5.44 (2.89) [1]<br>4.21-98.52 (16.82) [2]	n.d.-77.0 (12.57) [1]<br>0.3-5.36 (2.61) [2]	This study
Pearl River Delta, China	0.017-285.83 (6.57) [1]<br>0.017-320.50 (16.89) [2]	0.14-24.53 (6.25) [1]<br>0.11-26.48 (7.86)[2]	-	10
Bohai coastal region, China	0.40-12.78 (3.09)	0.96-4534.41 (277.02)	<0.63-79.61 (7.99)	11
Yangtze River, China	n.d.-3.06 (0.70)	0.52-18.0 (9.55)	n.d.-41.9 (7.68)	12
Huai River Basin	4.7	18	-	13
Hanjiang River, China	51.8	81	-	14
Haihe River, China	2.0-7.6	4.4-42	-	15
Taihu Lake, China	19	15	-	13
River Rhine watershed, Netherland	1.34-24.8	2.13-34.1	2.84-163	16
Orge River, France	17.4	9.4	4.4	17
River Elbe, Germany	0.5-2.9 (1.62)	2.8-9.6 (6.36)	0.09-3.4 (2.13)	18
Hyogo prefecture coastal area, Japan	n.d.-97	n.d.-360	-	19
Multiple rivers and lakes in the United State	0.1-254	0.03-43	n.d.-0.88	20
Three rivers in South Africa	n.d.-181.8	1.7-314.4	-	21

1 represents high water period

2 represents low water period

Values in parentheses indicate arithmetic mean concentration

Table S7 Component Score Coefficient Matrix of Principal component analysis. (a) HWP; (b) LWP.

Component Score Coefficient Matrix (a)

	**Component**		
	1	2	3
PFBA	0.136	-0.19	0.295
PFPeA	0.239	-0.195	0.006
PFHxA	0.21	-0.025	-0.027
PFHpA	0.248	-0.043	-0.115
PFOA	0.187	0.172	-0.197
PFNA	0.181	0.122	-0.063
PFBS	-0.145	0.073	0.797
PFOS	-0.117	0.61	0.259
F53B	0.059	-0.545	0.118

Extraction Method: Principal Component Analysis.
Rotation Method: Varimax with Kaiser Normalization

Component Score Coefficient Matrix (b)

	**Component**		
	1	2	3
PFBA	0.243	-0.140	-0.113
PFPeA	0.312	0.026	-0.138
PFHxA	0.275	0.083	0.244
PFHpA	0.347	-0.015	0.058
PFOA	0.069	-0.028	0.582
PFNA	0.124	0.099	-0.468
PFBS	-0.009	-0.171	0.011
PFOS	-0.025	0.452	-0.102
F53B	-0.055	0.448	-0.081

Extraction Method: Principal Component Analysis.
Rotation Method: Varimax with Kaiser Normalization

Table S8 Survey of Beijing industrial enterprises and PFCs related enterprises (2015)

Sector	Enterprise Amount	Total industrial output value[1]	PFCs related
Coal Mining and Dressing	4	5,491,992	N
Petroleum and Natural Gas Extraction	2	***	N
Ferrous Metals Mining and Dressing	7	1,316,681	N
Nonmetal Minerals Mining and Dressing	5	23,348	N
Other Mining and Dressing	6	2,029,296	N
Farm Byproducts Processing	144	3,825,018	Y
Food Production	125	2,789,079	Y
Beverage Production	42	1,919,525	Y
Tobacco Processing	1	***	Y
Textile Industry	27	206,051	Y
Garments, Shoes and Hats Production	141	1,379,236	Y
Leather, Furs, Down and Related Products	15	111,189	Y
Timber Processing, Bamboo, Cane, Palm Fiber and Straw Products	16	135,018	Y
Furniture Manufacturing	70	798,476	Y
Papermaking and Paper Products	41	644,423	Y
Printing and Record Medium Reproduction	118	1,230,727	Y
Cultural, Educational and Sports Goods	35	830,143	Y
Petroleum Processing, Coking and Nuclear Fuel Processing	23	8,453,806	N
Raw Chemical Materials and Chemical Products	215	3,520,728	N
Medical and Pharmaceutical Products	193	6,690,347	Y
Chemical Fiber	3	***	N
Rubber and Plastic Products	124	1,128,322	N
Nonmetal Mineral Products	251	4,880,385	N
Smelting and Pressing of Ferrous Metals	26	1,338,253	N
Smelting and Pressing of Nonferrous Metals	38	686,892	N
Metal Products	228	3,062,268	Y
Ordinary Machinery Manufacturing	252	5,505,967	N
For Special Purposes Equipment Manufacturing	329	5,903,160	N
Automobile manufacturing	237	36,476,369	N
Transport Equipment Manufacturing	74	3,815,408	N
Electric Equipment and Machinery	267	7,375,878	Y
Telecommunication Equipment, Computer and Other Electronic Equipment	299	24,244,643	N

Instruments, Meters, Cultural and Office Machinery	176	2,573,411	N
Metal products machinery and equipment repair	15	382,012	Y
Handicraft Articles and Other Manufacture	24	523,436	N
Reclaiming and Processing of Abandoned Resource and Waste Material	8	76,471	N
Production and Supply of Electric Power and Heat	63	40,869,661	N
Production and Supply of Gas	22	2,988,642	N
Production and Supply of Tap Water	20	613,411	N
Total	3,686	183,839,672	

1. the unit of total industrial output value is $\times 10^4$ Yuan.

2. *** means the data is not revealed.

3. Y means that there is no obvious evidence.

4. N means that there is obvious evidence.

Table S9 The WWTPs with over 80,000 m^3/d treatment capability in North Canal Basin

District	WWTP	Treatment process	Attended time	Treatment capacity thousand m^3/d
Chaoyang	Beixiaohe	A/A/O and MBR	1990	100
	Jiuxianqiao	Oxidation ditch	2000	200
	Gaobeidian	Active sludge	1999	1000
	Xiaohongmen	Active sludge	2006	600
Shunyi	Capital air port	Oxidation ditch	2009	90
	Shunyi district	Oxidation ditch	2007	80
shijingshan	lugouqiao	A/A/O	2004	100
Tongzhou	Beishui	secondary biochemical treatment	2005	100
Haidian	Qinghe	Active sludge	2003	400
Daxing	Xingshui	Oxidation ditch	2000	80
Fengtai	Wujiacun	Active sludge	2003	80

WWTPs with a treatment capacity over 100 thousand m^3/d are displayed in Fig. 1.

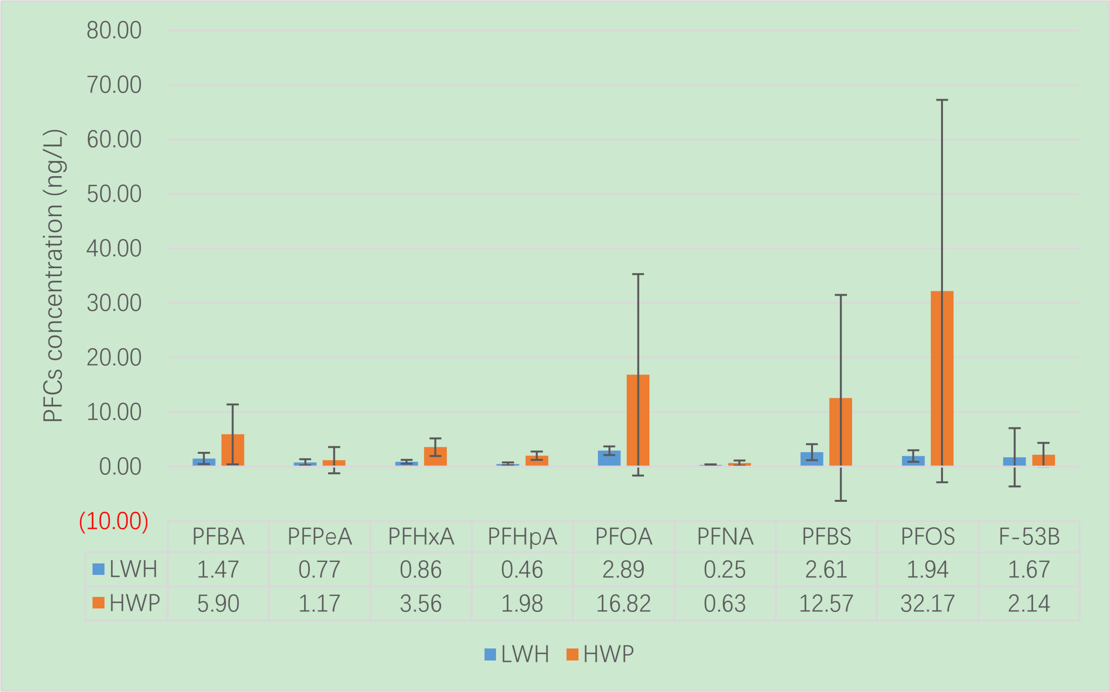

Fig. S1 the average concentration of PFCs in different water periods

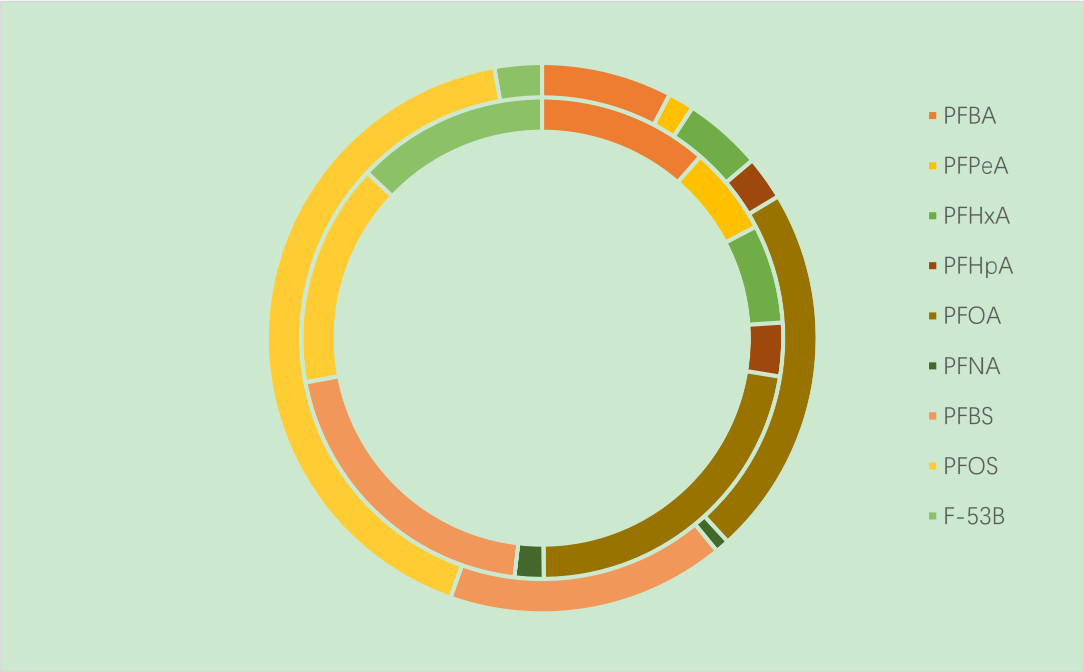

Fig. S2 Proportions of the average concentration of PFCs in North Canal Basin; the outside circle depicts the distribution in LWP sampling; the inner circle describes the distribution in HWP sampling.

References

1. Beijing water authority. Rainfall regime statistics. *Beijing Water Authority Network* http://hdsw.bjhd.gov.cn/zxfw/bmcxfw/hdqyqcx/index_2.htm. (Accessed: 18th December 2015) (2015).

2. Zhang, L. *et al*. The Inventory of Sources, Environmental Releases and Risk Assessment for Perfluorooctane Sulfonate in China. *Environ Pollut*. **165**, 193-198 (2012).

3. Wang, Z., Cousins, I. T., Scheringer, M., Buck, R. C. & Hungerbühler, K. Global Emission Inventories for C4–C14 Perfluoroalkyl Carboxylic Acid (Pfca) Homologues From 1951 to 2030, Part I: Production and Emissions From Quantifiable Sources. *Environ Int*. **70**, 62-75 (2014).

4. Wang, S. *et al*. First Report of a Chinese Pfos Alternative Overlooked for 30 Years: Its Toxicity, Persistence, and Presence in the Environment. *Environ Sci Technol*. **47**, 10163–10170 (2013).

5. Xiao, F., Halbach, T. R., Simcik, M. F. & Gulliver, J. S. Input Characterization of Perfluoroalkyl Substances in Wastewater Treatment Plants: Source Discrimination by Exploratory Data Analysis. *Water Res*. **46**, 3101-3109 (2012).

6. Ma, R. *et al*. Characterization of Pharmaceutically Active Compounds in Beijing, China: Occurrence Pattern, Spatiotemporal Distribution and its Environmental Implication . *J Hazard Mater*. In press (2016).

7. Hoke, R. A., Bouchelle, L. D., Ferrell, B. D. & Buck, R. C. Comparative Acute Freshwater Hazard Assessment and Preliminary Pnec Development for Eight Fluorinated Acids. *Chemosphere*. **87**, 725-733 (2012).

8. Lin, A. Y., Panchangam, S. C. & Ciou, P. High Levels of Perfluorochemicals in Taiwan’S Wastewater Treatment Plants and Downstream Rivers Pose Great Risk to Local Aquatic Ecosystems. *Chemosphere*. **80**, 1167-1174 (2010).

9. Zushi, Y., Hogarh, J. N. & Masunaga, S. Progress and Perspective of Perfluorinated Compound Risk Assessment and Management in Various Countries and Institutes. *Clean Technol Envir*. **14**, 9-20 (2012).

10. Pan, C. *et al*. Contamination Profiles of Perfluoroalkyl Substances in Five Typical Rivers of the Pearl River Delta Region, South China. *Chemosphere*. **114**, 16-25 (2014).

11. Wang, P. *et al*. Occurrence and Transport of 17 Perfluoroalkyl Acids in 12 Coastal Rivers in South Bohai Coastal Region of China with Concentrated Fluoropolymer Facilities. *Environ Pollut*. **190**, 115-122 (2014).

12. Pan, C. *et al*. Spatiotemporal Distribution and Mass Loadings of Perfluoroalkyl Substances in the Yangtze River of China. *Sci Total Environ*. **493**, 580-587 (2014).

13. Yu, N. *et al*. Occurrence of Perfluoroalkyl Acids Including Perfluorooctane Sulfonate Isomers in Huai River Basin and Taihu Lake in Jiangsu Province, China. *Environ Sci Technol*. **47**, 710-717 (2013).

14. Wang, B. *et al*. Distribution of Perfluorinated Compounds in Surface Water From Hanjiang River in Wuhan, China. *Chemosphere*. **93**, 468-473 (2013).

15. Li, F. *et al*. Perfluorinated Compounds in Haihe River and Dagu Drainage Canal in Tianjin, China. *Chemosphere*. **84**, 265-271 (2011).

16. Möller, A. *et al*. Distribution and Sources of Polyfluoroalkyl Substances (Pfas) in the River Rhine Watershed. *Environ Pollut*. **158**, 3243-3250 (2010).

17. Labadie, P. & Chevreuil, M. Partitioning Behaviour of Perfluorinated Alkyl Contaminants Between Water, Sediment and Fish in the Orge River (Nearby Paris, France). *Environ Pollut*. **159**, 391-397 (2011).

18. Ahrens, L., Felizeter, S., Sturm, R., Xie, Z. & Ebinghaus, R. Polyfluorinated Compounds in Waste Water Treatment Plant Effluents and Surface Waters Along the River Elbe, Germany. *Mar Pollut Bull*. **58**, 1326-1333 (2009).

19. Takemine, S. *et al*. Discharge of Perfluorinated Compounds From Rivers and their Influence On the Coastal Seas of Hyogo Prefecture, Japan. *Environ Pollut*. **184**, 397-404 (2014).

20. Flores, C., Ventura, F., Martin-Alonso, J. & Caixach, J. Occurrence of Perfluorooctane Sulfonate (Pfos) and Perfluorooctanoate (Pfoa) in N.E. Spanish Surface Waters and their Removal in a Drinking Water Treatment Plant that Combines Conventional and Advanced Treatments in Parallel Lines. *Sci Total Environ*. **461-462**, 618-626 (2013).

21. Mudumbi, J. B. N., Ntwampe, S. K. O., Muganza, F. M. & Okonkwo, J. O. Perfluorooctanoate and Perfluorooctane Sulfonate in South African River Water. *Water Science & Technology*. **69**, 185-194 (2014).
